# Examination of mechanisms underlying enhanced memory performance in action video game players: a pilot study

**DOI:** 10.3389/fpsyg.2015.00843

**Published:** 2015-06-16

**Authors:** Xianchun Li, Xiaojun Cheng, Jiaying Li, Yafeng Pan, Yi Hu, Yixuan Ku

**Affiliations:** ^1^Key Laboratory of Brain Functional Genomics, Ministry of Education, Shanghai Key Laboratory of Brain Functional Genomics, School of Psychology and Cognitive Science, East China Normal University, ShanghaiChina; ^2^Department of Neurology, Physiology and Psychiatry, University of California, San Francisco, San Francisco, CAUSA

**Keywords:** change detection, action video game, partial-report procedure, memory decay, retro-cue

## Abstract

Previous studies have shown enhanced memory performance resulting from extensive action video game playing. The mechanisms underlying the cognitive benefit were investigated in the current study. We presented two types of retro-cues, with variable intervals to memory array (Task 1) or test array (Task 2), during the retention interval in a change detection task. In Task 1, action video game players demonstrated steady performance while non-action video game players showed decreased performance as cues occurred later, indicating their performance difference increased as the cue-to-memory-array intervals became longer. In Task 2, both participant groups increased their performance at similar rates as cues presented later, implying the performance difference in two groups were irrespective of the test-array-to-cue intervals. These findings suggested that memory benefit from game plays is not attributable to the higher ability of overcoming interference from the test array, but to the interactions between the two processes of protection from decay and resistance from interference, or from alternative hypotheses. Implications for future studies were discussed.

## Introduction

Video games are ubiquitous among today’s generation. A great body of literature has revealed that action video game players (AVGPs), relative to non-video game players (NVGPs), reap broad cognitive benefits from extensive action video game playing. These benefits included visual acuity (Green and Bavelier, 2007; Granic et al., 2014), attention flexibility (Green and Bavelier, 2003), stimulus-response mapping (Clark et al., 1987; Castel et al., 2005), encoding speed (Wilms et al., 2013), and executive functioning (Strobach et al., 2012). Extensive experience playing action video games can even affect memory for the stimuli presented in a very short period (e.g., iconic memory and visual working memory), resulting in better accuracy (Boot et al., 2008; Blacker and Curby, 2013), higher precision (Sungur and Boduroglu, 2012), and more efficient strategy in retrieving information (Clark et al., 2011). In the current study, we focused on the mechanisms underlying such improved memory performance.

There was a preliminary study on the mechanisms for memory advantage in AVGPs over NVGPs, through the change detection paradigm (Blacker and Curby, 2013). In the paradigm, a *memory array* of several objects presented briefly, followed by a short *retention interval* and then a *test array*. Observers need to report whether objects in the test array are identical to those in the memory array. The paradigm has been extensively adopted to estimate memory capacity for the fast presented stimuli (Luck and Vogel, 1997; Luria et al., 2010; Blacker et al., 2014; Qi et al., 2014). In Blacker and Curby’s (2013) study, they manipulated the presentation time of memory array (168 ms vs. 1018 ms). Their results showed that this manipulation did not affect the performance difference between AVGPs and NVGPs, indicating that encoding duration was not a possible factor on the advantage of AVGPs over NVGPs.

In line with this study, we would examine retained information after encoding, by presenting cues during the retention interval (i.e., retro-cue) in the change detection task (Appelbaum et al., 2013). In this partial-report procedure, a retro-cue indicates the to-be-compared item in the memory array. Observers need to judge whether it is matched to the one in the test array, without consideration of other un-cued items. Previous studies have revealed that the partial-report procedure compared with the whole-report procedure can detect higher memory performance (Landman et al., 2003; Makovski et al., 2008). This was explained that in the whole-report procedure there would be interference from similar items in the memory array (Luria et al., 2010) or on previous trials (namely proactive interference; Lin and Luck, 2012), while it is not in the partial-report procedure.

Growing evidence indicated that time-related decay might be the most likely source of forgetting on the change detection task (Barrouillet et al., 2007; Matsukura et al., 2007; Portrat et al., 2008; Barrouillet and Camos, 2012). For example, recall performance decreased in longer retention intervals (Zhang and Luck, 2009; Woodman et al., 2012; Morey and Bieler, 2013). One previous study, with the arrangement of variable delays of cue to memory array, revealed that detection accuracy decreased as the delay became longer (Becker et al., 2000). There are other possibilities than the decay hypothesis, such as the interference hypothesis, for the decreased performance in change detection task. Some studies have demonstrated that retained information after encoding could be interfered by the subsequent new stimuli (Landman et al., 2003; Makovski et al., 2008). For example, a recent study with the adjustment of delays of test array to cue found that the shorter delay indicating larger interference was associated with the decreased memory accuracy (Pertzov et al., 2013).

Based on these hypotheses, we proposed that the memory advantage in AVGPs over NVGPs might be from their higher abilities of protecting information against decay and/or interference. These were examined through two partial-report procedures that were originally used in former studies. One was the normal retro-cue procedure (Task 1), with the arrangement of variable delays of cue to memory array and of constant interval between memory array and test array (Landman et al., 2003; Matsukura et al., 2007; Makovski et al., 2008). The other was to vary delays of test array to cue (Task 2), while keeping the delay of cue to memory array constant (Becker et al., 2000; Pertzov et al., 2013). Note that the present study was not aimed to explore the mechanisms for retro-cue effects in the change detection task. Rather, we were interested in whether the memory advantage of AVGPs over NVGPs could be explained by the decay hypothesis and/or the interference hypothesis.

## Materials and Methods

### Participants

Thirty-nine male undergraduate and graduate students from East China Normal University, with normal or corrected-to-normal vision, were recruited. Following previous studies (Green and Bavelier, 2003, 2006, 2007; Blacker and Curby, 2013), AVGPs (*n* = 21, age = 20.1 ± 2.7 years) had played action video games at least 4 days per week over the past 6 months, with a minimum of 1 h per day. The games included *Counter-Strike, Call of Duty, Assassin’s Creed, Resident Evil, Cross Fire, Left 4 Dead, NBA2K13, Soul Sacrifice, and Dragon Nest*. NVGPs (*n* = 18, age = 20.8 ± 2.4 years) had little action video-game plays in the past half year. Written informed consents were attained from all participants and they were compensated for their participation. This study was approved by the Institutional Review Board of East China Normal University.

### Stimuli and Procedures

Stimuli were presented on a 17-inch LCD monitor (SONY, 96 dpi, refresh rate: 60 Hz). The distance between participants and the screen was about 50 cm. There were two tasks (Task 1 and Task 2) × 5 blocks × 36 trials. The order of two tasks was counter-balanced among participants. Trials in a block and blocks in a task were randomly arranged. Twelve practice trials were provided to familiarize participants with the experimental procedures. Feedbacks were presented in practice but not in formal trials. The procedure of performing a trial was described as follows.

#### Task 1: Varying the Delay of Cue to the Offset of Memory Array

**FIGURE 1:**
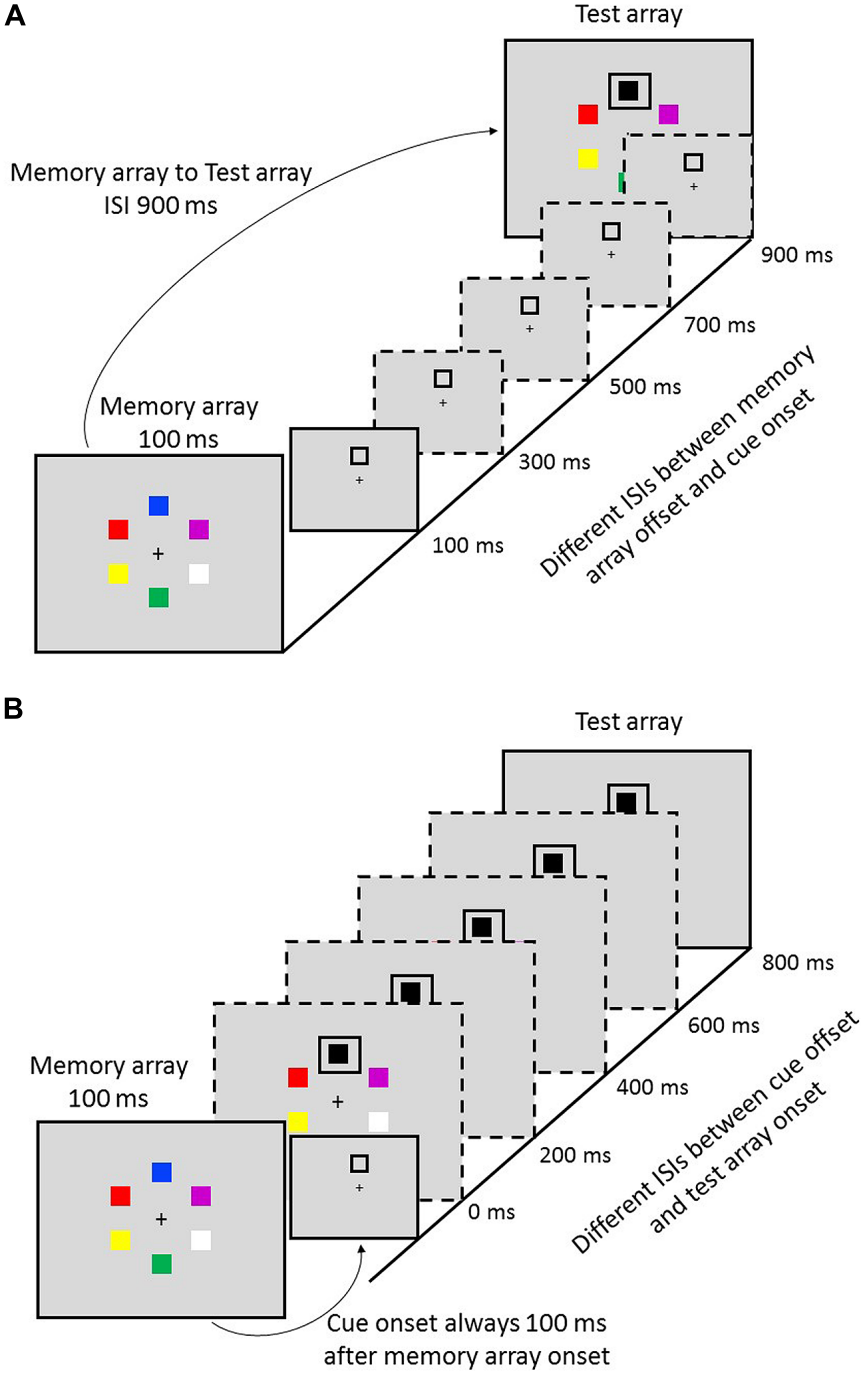
**The partial-report procedure of two tasks. (A)** Task 1: variable cue-to-memory-array delays. **(B)** Task 2: variable test-array-to-cue delays.

Participants were instructed to detect whether there is a change between the memory array and the test array in a change detection task. Each trial began with a 1000-ms central fixation (black, 0.4° diameter), followed by a memory array (presented for 100 ms) of six different-colored squares (1.6° × 1.6°). The squares equally spaced around a clock face (diameter: 8.6°). Each color of squares was randomly selected, without replacement, from a set of seven colors (i.e., black, white, red, yellow, blue, green, and purple). After the offset of the memory array, it was the retention interval (900 ms) with a cue inserted. The cue was a black grid appeared at one of the six positions in the memory array, indicating the location to be tested with 100% validity. Half trials were with the change, and half were not. The cue-to-memory-array delay was varied from 100 to 900 ms, with a step of 200 ms (see **Figure 1A**). After the retention interval, a test array was presented. Participants clicked the square with a mouse if they thought there was a change. Otherwise, they pressed the space bar. After the response, one trial ended.

#### Task 2: Varying the Delay of the Onset of Test Array to Cue

Task 2 was similar to Task 1, except that the cue was always presented 100 ms after the offset of memory array and the delays of the onset of test array to cue varied from 0 to 800 ms with a step of 200 ms (see **Figure 1B**).

### Data Collection and Analysis

The dependent variable was the accuracy of memory performance, indicated by the ratio of correct responses to the total number of trials tested in each condition, and the independent variables were participant groups (AVGPs vs. NVGPs) and delays in the two tasks. We excluded one AVGP’s data in Task 1 due to software malfunction during experiment. Analyses of variance (ANOVAs), regression models, and *t*-tests were conducted to investigate the differential time courses of memory retention between two groups.

## Results

**Table 1** described the accuracy of performance at the five delays in Task 1 and Task 2. The main interests were the performance differences between two groups (AVGPs vs. NVGPs) and the moderating effect of experimental manipulation (i.e., the arranged delays).

**Table 1:** The accuracy of memory performance in two groups (*M* ± SD).

	Action video game players (AVGPs)	Non-action video game players (NVGPs)
Task 1: The delay of cue to memory array at:		
100 ms	0.87 ± 0.09	0.84 ± 0.09
300 ms	0.90 ± 0.07	0.79 ± 0.09
500 ms	0.87 ± 0.10	0.80 ± 0.08
700 ms	0.85 ± 0.10	0.74 ± 0.09
900 ms	0.85 ± 0.10	0.76 ± 0.08
Task 2: The delay of test array to cue at:		
0 ms	0.84 ± 0.11	0.72 ± 0.12
200 ms	0.84 ± 0.08	0.73 ± 0.11
400 ms	0.88 ± 0.09	0.76 ± 0.09
600 ms	0.88 ± 0.07	0.79 ± 0.09
800 ms	0.88 ± 0.08	0.77 ± 0.11

In Task 1, a mixed-design ANOVA was conducted with a between-subject factor of group (AVGPs vs. NVGPs) and a within-subject factor of cue-to-memory-array delay. The results revealed the significant main effects of group, *F*(1,36) = 13.43, *p* < 0.01, $\eta_{p}^{2}$ = 0.27, with AVGPs performed better than NVGPs, and delay, *F*(4,144) = 6.47, *p* < 0.001, $\eta_{p}^{2}$ = 0.15. The interaction between group and delay was also significant, *F*(4,144) = 2.77, *p* < 0.05, $\eta_{p}^{2}$ = 0.07, indicating that the performance differences in AVGPs vs. NVGPs were modulated by the lapse of time.

**FIGURE 2:**
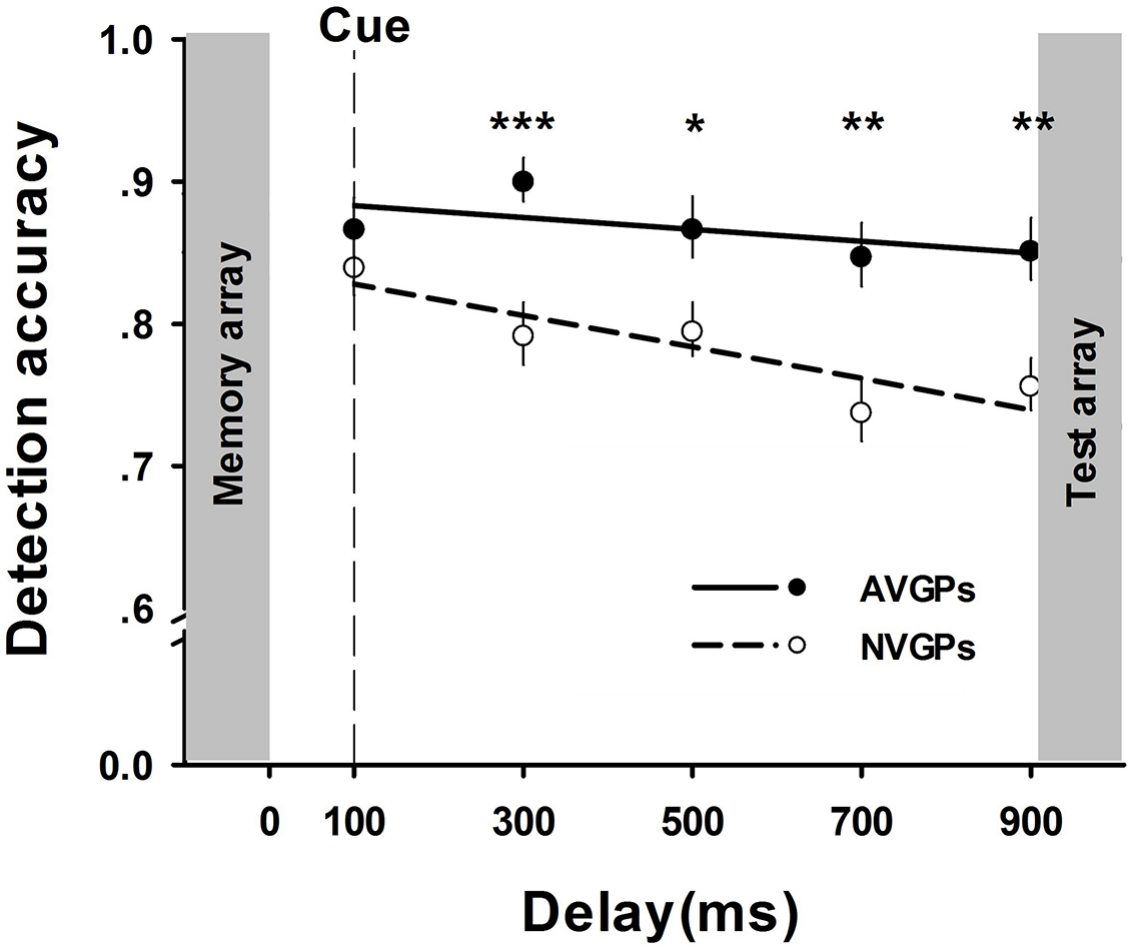
**The accuracy of memory performance (means and SE) across cue-to-memory-array delays for action video game players (AVGPs) and non-action video game players (NVGPs) in Task 1.** Group differences were demonstrated at five delays except for the 100 ms condition. *p < 0.05, **p < 0.01, ***p < 0.001.

To better understand the overall trend of time-based performance, accuracy slopes were estimated by linear regressions of accuracy across delays (Pertzov et al., 2013). The decreasing rate was significant from zero in NVGPs, β = -0.34, *p* < 0.001, but not in AVGPs, β = -0.13, *p* > 0.05. These results indicated that retained visual information in memory markedly deteriorated in NVGPs but not in AVGPs. To observe when the memory advantage of AVGPs occurred, we conducted a series of *t*-tests at all five delay conditions. The results showed significant differences of performance between AVGPs and NVGPs at delays longer than 100 ms, all *t*s > 2.48, *p*s < 0.05, *d*s > 0.81, but not for the shortest delay (100 ms), *t* (36) = 0.92, *p* > 0.05, *d* = 0.30 (see **Figure 2**).

**FIGURE 3:**
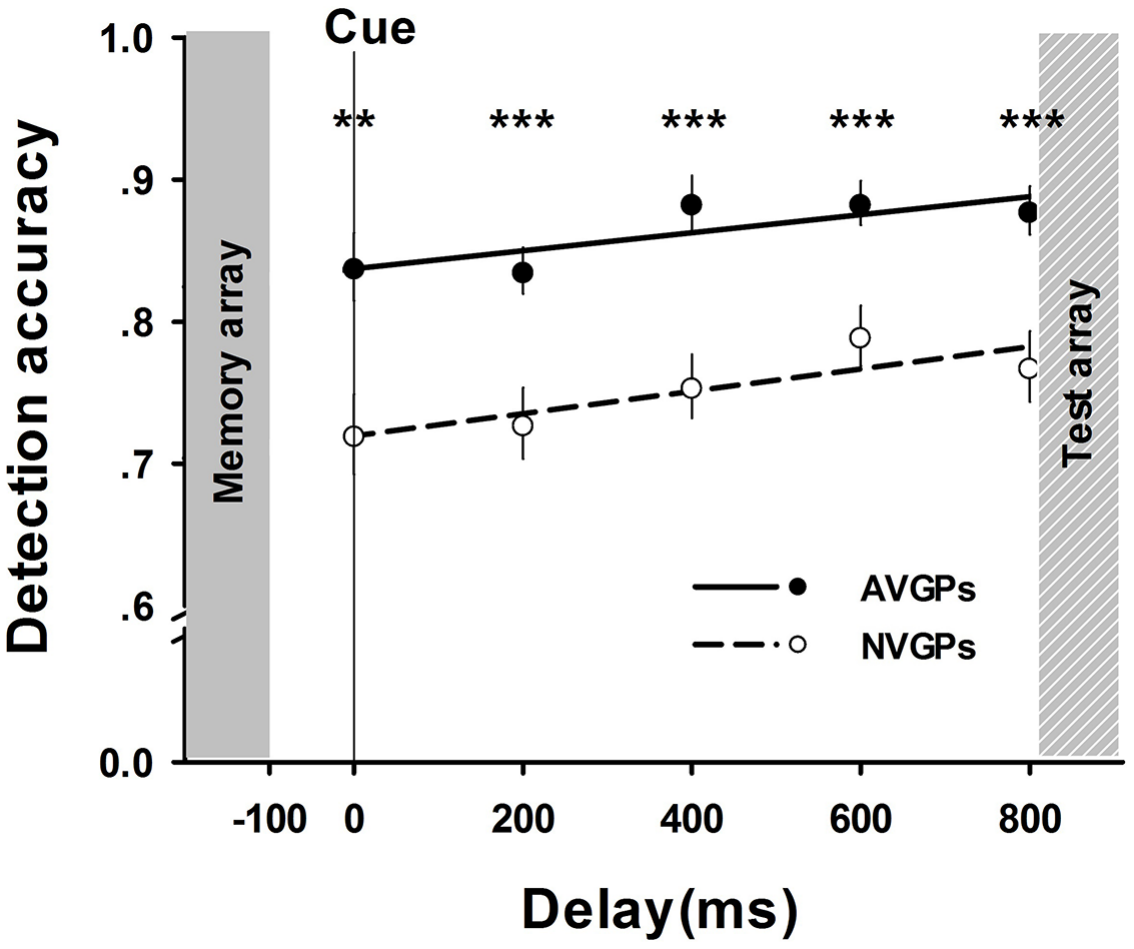
**The accuracy of memory performance (means and SE) across test-array-to-cue delays in Task 2.** Group differences were observed in all delays. **p < 0.01, ***p < 0.001.

In Task 2, a mixed-design ANOVA was performed with a between-subject factor of group (AVGPs vs. NVGPs) and a within-subject factor of test-array-to-cue delay. Consistent with the results in Task 1, there were significant main effects for group, *F* (1,37) = 23.81, *p* < 0.001, $\eta_{p}^{2}$ = 0.39, and delay, *F*(4,144) = 5.40, *p* = 0.001, *$\eta_{p}^{2}$ = 0.13*. However, the interaction was not significant. Furthermore, the analyses of accuracy slopes revealed that the memory performance of both AVGPs and NVGPs increased as delays increased (i.e., interference decreased) at similar rates (NVGPs: β = 0.21, *p* < 0.05; AVGPs: β = 0.21, *p* < 0.05). Subsequent *t*-tests results suggested that differences between both groups were marked at all conditions of delay, *p*s < 0.01 (see **Figure 3**). These results implied that the performance difference between two groups could not be influenced by the amount of interference.

## Discussion

Previous studies have demonstrated that extensive exposure to action video game playing can enhance memory performance, and the enhancement was not attributed to the length of time period for encoding (Clark et al., 2011; Blacker and Curby, 2013; Blacker et al., 2014). In line with those studies, the present study explored the mechanisms underlying the memory advantage in AVGPs over NVGPs through the partial-report procedure of a change detection paradigm. We arranged retro-cues during the retention interval, to examine the time course of information retained in memory and the possible influence from the following test array.

In Task 1, the interval between the offset of the memory array and the onset of the test array was fixed at 900 ms while cue-to-memory-array intervals varied from 100 to 900 ms, which involves the processing periods for iconic memory (<500 ms; e.g., Griffin and Nobre, 2003; Persuh et al., 2012; Clarke and Mack, 2014) and visual working memory (500–1000 ms; e.g., Luck and Vogel, 1997; Luck and Hollingworth, 2008). We first observed the memory advantage in AVGPs for all conditions except the shortest interval (i.e., 100 ms). In Task 2, the cue-to-memory-array interval was fixed at 100 ms, when iconic memory occurs. Meanwhile, the test-array-to-cue interval was variable from 0to 800 ms. And in this situation, the memory advantage in AVGPs maintained at all conditions. Similar to our findings, previous studies demonstrated that AVGPs had better memory performance in other experimental tasks assessing iconic memory and working memory, such as the *N*-back tasks (Boot et al., 2008; Colzato et al., 2013), the enumeration task (Green and Bavelier, 2006), the temporal order recall task (Green and Bavelier, 2006; West et al., 2008), the franker task (Cain et al., 2012), and the multiple identity tracking task or color wheel task (Sungur and Boduroglu, 2012). All these findings indicated the benefits from playing action video games for the memory of briefly presented stimuli.

It was notable that the amount of information maintained in retention interval changed with time in this study. As the interval became longer in Task 1, NVGPs showed decreased performance. The decreasing trend was similar to the finding in a previous study (Becker et al., 2000). In that study, the interval between a memory array and a test array was short (281 ms) and the retro-cue appeared at variable delays from the memory array (from 16, 82, 149, 215, to 281 ms). Participants’ accuracy decreased gradually as the delay increased. Different from the performance of NVGPs in the current study and in Becker et al.’s (2000) study, AVGPs in the present study showed stable performance across all delays, indicating that their memory was not affected by the time lapse.

However, it is difficult to dissociate the causes of these effects. As the cue-to-memory-array interval and the test-array-to-cue interval were both varied in Task 1, both the memory decay across time and the interference from the test array could potentially account for the observed effects. The performance difference between AVGPs and NVGPs could be explained by both decay hypothesis and interference hypothesis. However, the later hypothesis could not account for our results in Task 2. Here, both AVGPs and NVGPs increased their performance as the test-array-to-cue interval became longer. More specifically, the performance difference between groups was stable, irrespective of test-array-to-cue interval (Appelbaum et al., 2013). Therefore, we proposed that the performance advantage in AVGPs relative to NVGPs could not be attributed to their better ability to overcome interference from the test array in our current settings. This proposal did not mean that the decay hypothesis would be a better theory. We thus test the decay hypothesis alone with an additional tentative experiment.

We recruited additional 17 participants to examine the situation when only the cue-to-memory-array interval was manipulated (see Supplemental Materials). The experimental task was similar to task 1 with following exceptions: (1) a mask stimulus inserted after the offset of memory array and presented for 100 ms; (2) a fixed interval from the test array to cue (160 ms); (3) variable intervals of cue to mask (from 0 to -800 ms, with the step of 160 ms). The experimental arrangement allowed us to examine how the retained information in memory changed with time while the interference from subsequent stimuli kept constant. The preliminary results showed a significant main effect of group. However, neither delay nor the interaction was significant. This indicated that performance advantage in AVGPs was not affected by the lapse of time when the test-array-to-cue interval kept fixed. Altogether, either decay hypothesis or interference hypothesis alone could not account for the AVGPs advantage, but the interaction between them might do, when both the cue-to-memory-array and the test-array-to-cue intervals were manipulated simultaneously (as in Task 1). It could also be possible that alternative hypotheses affected both decay and interference. Therefore, the investigation of potential mechanism for the better memory performance in AVGPs compared with NVGPs should be carefully explored in future studies, with focus on iconic memory or working memory, respectively.

## Conclusion

The present study explored potential mechanisms for the previously observed memory benefit from playing games. Our findings suggested that enhanced memory in AVGPs compared with NVGPs could not be from overcoming interference from following stimuli. There might be other possibilities than memory decay or interference hypothesis. There might be other possibilities than memory decay or interference hypothesis that needed to be explored in the future, such as encoding speed (Wilms et al., 2013), visual sensitivity (Appelbaum et al., 2013), attention (Boot et al., 2008), strategy (Clark et al., 2011), or executive control (Boot et al., 2008). Additionally, given that expert-novice comparison could not interpret the causal role of experiences in cognitive benefits, carefully controlled intervention studies are would be critical (Green and Bavelier, 2012; Bejjanki et al., 2014). Whether the benefits can be generalized to other domains, such as academic achievement, can also be explored in the future.

## Conflict of Interest Statement

The authors declare that the research was conducted in the absence of any commercial or financial relationships that could be construed as a potential conflict of interest.
