# Supplementary material for: Examination of mechanisms underlying enhanced memory performance in action video game players: a pilot study
Source: Front Psychol. 2015 Jun 16;6:843. doi: 10.3389/fpsyg.2015.00843 (PMC4468821; doi:10.3389/fpsyg.2015.00843)
Supplement: Supplementary file 1 [file Table_1.DOC]

***Supplemental Materials***

**Examination of Mechanisms Underlying Enhanced Memory Performance in Action Video Game Players: A Pilot Study**

**Xianchun Li^1#^, Xiaojun Cheng^1#^, Jiaying Li^1^, Yafeng Pan^1^, Yi Hu^1^, Yixuan Ku^1, 2^***

^1^ Key Laboratory of Brain Functional Genomics, Ministry of Education, Shanghai Key Laboratory of Brain Functional Genomics, School of Psychology and Cognitive Science, East China Normal University Shanghai, China.

^2^ Departments of Neurology, Physiology and Psychiatry, University of California, San Francisco, San Francisco, USA.

^﹟^These two authors contributed equally to this work and could been seen as co-first authors.

*** Correspondence to:** Yixuan Ku: [yixuanku@gmail.com](mailto:yixuanku@gmail.com)

1. **Supplementary Data**

**1.1. Methods**

**1.1.1. Participants**

The current experiment involved 17 individuals. Among them, participants were classified as AVGPs (n = 7; age = 23.57 ± 2.64 yr; all males) if they played action video games with a minimum of 4 days per week over the past 6 months (> 1 hr/day). Individuals were identified as NVGPs (n = 10; age = 22.70 ± 1.49 yr; six males) if they did not play action video games in the past half year. All of them gave written informed consents and were compensated for participation. This study was approved by the Institutional Review Board of East China Normal University.

**1.1.2. Procedures**

The experimental procedure (**Supplementary Figure 1**) included five blocks, each involving 36 trials. The experimental task was similar to task 1 with following exceptions: 1) a mask stimulus inserted after the offset of memory array and presented for 100 ms; 2) a fixed interval from the retro-cue to the onset of test array (160 ms); 3) variable delays from the mask to the retro-cue (from 0-800 ms, with a step of 160 ms). The mask stimuli consisted of six same mosaic squares (1.6º × 1.6º), which were equally placed around a clock face (diameter: 8.6º).

**1.2. Results**

The accuracy of performance were presented in **Supplementary Table 1**.

A 2 (group: AVGPs vs. NVGPs) × 5 (cue-to-mask delay: 0/160/320/640/800 ms) mixed ANOVA revealed a significant main effect of group, *F* (1, 15) = 6.30, *p* < 0.05, *η*_patial_^2^ = 0.30, with AVGPs (0.71 ± 0.04) performed better than NVGPs (0.60 ± 0.03). However, neither the main effect of cue-to-mask delay nor the group × delay interaction failed to reach significance, *p*s > 0.19.

**2. Supplementary Figures and Tables**

## 2.1. Supplementary Tables

**Supplementary Table 1.** The accuracy of performance in the tentative study (*M* ± *SD*)

|  | AVGPs |  | NVGPs |
| --- | --- | --- | --- |
| *The delay of cue to mask at:* | | | |
| 0 ms | 0.65 ± 0.14 |  | 0.56 ± 0.10 |
| 160 ms | 0.75 ± 0.17 |  | 0.60 ± 0.08 |
| 320 ms | 0.70 ± 0.15 |  | 0.64 ± 0.11 |
| 480 ms | 0.73 ± 0.11 |  | 0.62 ± 0.11 |
| 640 ms | 0.74 ± 0.12 |  | 0.58 ± 0.11 |

##
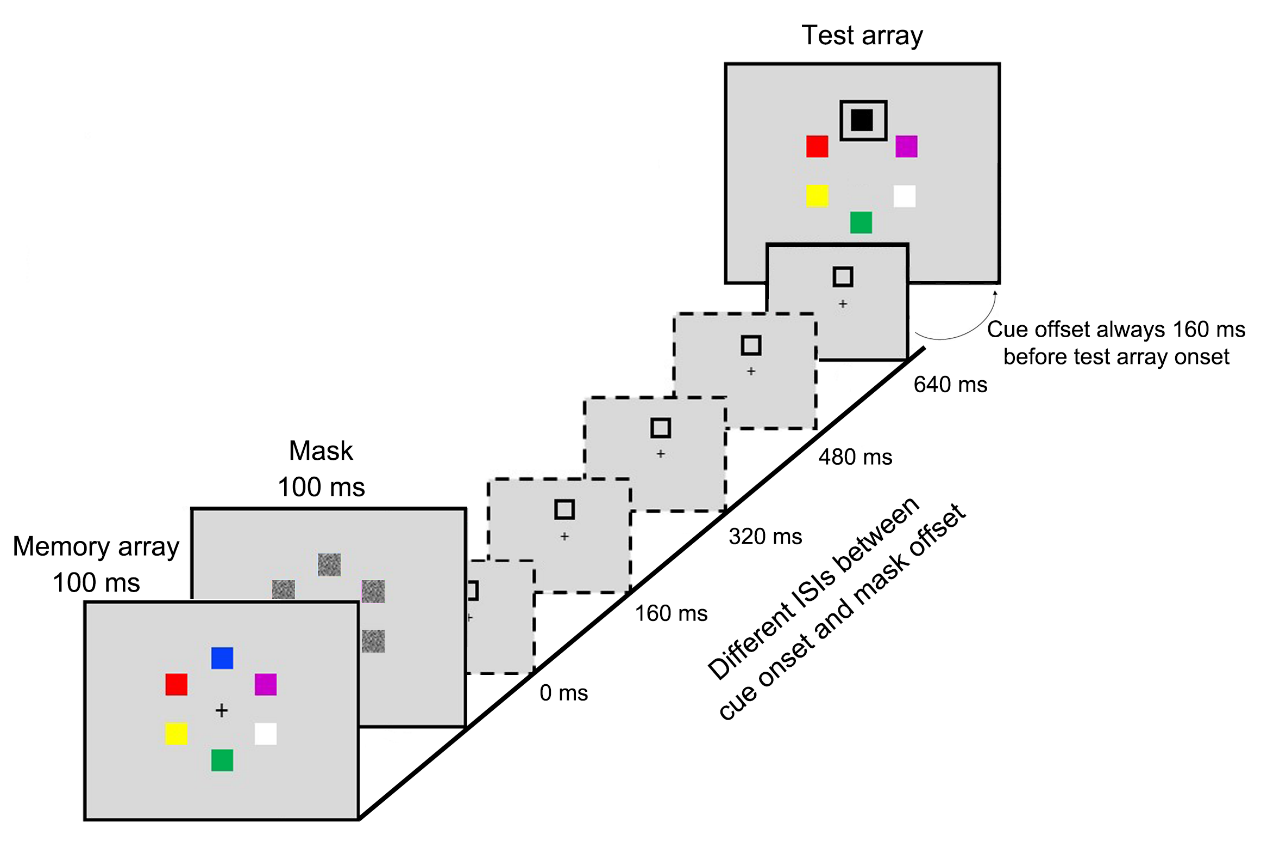
2.2. Supplementary Figures

**Supplementary Figure 1.** The tentative task: 1) variable durations from the mask to the retro-cue (0-800 ms, step: 160 ms); 2) a fixed interval between the retro-cue and the test-array (160 ms); 3) a mask (100 ms) inserted after the offset of memory array.
